# Empowering Patients: Promoting Patient Education and Health Literacy

**DOI:** 10.7759/cureus.27336

**Published:** 2022-07-27

**Authors:** Pradnya Brijmohan Bhattad, Luigi Pacifico

**Affiliations:** 1 Cardiovascular Medicine, Saint Vincent Hospital, University of Massachusetts Chan Medical School, Worcester, USA

**Keywords:** electronic health record, quality improvement, plan-do-study-act cycle, health literacy, patient education

## Abstract

Patients are generally keen to understand and obtain more information about their medical conditions. There exists a need to develop updated and thorough yet concise patient education handouts and to encourage healthcare providers (HCPs) to use uniform patient education methods.

A thorough review of literature on patient education material was performed prior to starting the study. A comparison with different resources regarding the appropriateness of patient education was done. Educating HCPs to effectively use patient educational materials incorporated into the electronic health record system, including electronic methods, such as the use of a patient portal, to help educate patients.

Strategies were formulated to reduce the amount of processing and attending time required for fetching appropriate materials and lead to fast, efficient, and effective patient education. To improve the physical and psychosocial wellbeing of a patient, personalized patient education handouts, in addition to verbal education by the HCPs, augment the betterment of patient care via shared decision making and by improving patient satisfaction and health literacy.

## Introduction

Patients are often eager to understand and know more about their medical conditions and health situation, and educating them with the most relevant, current, consistent, and updated information helps patients and their families significantly in the medical care and decision-making process [1]. 

Patients need formal education on the disease condition; they need to know their ailment, understand their symptoms, be educated on the diagnostics, appropriate medication use, and should be taught when to call for help. Several patient education handouts for various conditions are available, and there exists a need to assess which one is better suited for a particular disease/condition encountered and provides concise information. Patient education materials help educate the patients on their health conditions, improves their health literacy, and enhances and promotes informed decision-making based on the most current and updated medical and clinical evidence as well as patient preference [2].

Aims

The aim of this study was to develop updated patient education handouts and materials in addition to verbal counseling of the patients to help them understand the disease condition, diagnostic studies, proper advice on medications, and when to call for help. And to encourage healthcare providers (HCPs) to use uniform patient education materials.

Objectives

The objectives of this study are 1) the implementation of quality improvement techniques of Plan-Do-Study-Act (PDSA) cycles on patient education in clinical settings; 2) to enhance the delivery of patient education and create awareness amongst the HCPs regarding the importance of patient education and improved health literacy; 3) to verify if patient education handouts have the minimum necessary information that patient should know; 4) to compare patient education handouts from databases integrated in the electronic health record (EHR) with standard patient education database websites like the Centers for Disease Control and Prevention website, and MedlinePlus® site to make sure that they have the minimum necessary information; and 5) to educate and encourage HCPs on the use of appropriate patient education articles in the EHR and utilize an electronic patient portal for patient education, help transition the patient education to an electronic form, and increase efficacy and consistent patient education.

## Materials and methods

A comprehensive review of the patient education materials on the most common medical ailments in various clinical settings was performed. We compared the existing patient education database integrated in the EHR with the standard resources such as the CDC, MedlinePlus via retrospective chart study format to ensure the minimum necessary information is available. 

A comparison of existing educational material was completed by analyzing other patient education materials from resources such as UpToDate (the basics/beyond the basics), MedlinePlus, US National Library of Medicine of NIH, CDC, and the US Department of Health and Human Services to ensure that effective, most updated, current, and evidence-based information is provided to the patients from the educational materials.

Search words were incorporated to help search for the educational articles in the existing EHR by the title of the article. Educational materials studied were relevant to the common medical ailments in various clinical settings. The patient handouts were made available in such a way that these should be able to be sent either through an electronic patient portal or printed out.

HCPs were educated in a session with pre- and post-lecture survey qualitative and quantitative questionnaires. The impact of these interventions was further assessed by pre- and post-intervention surveys after educating the HCPs.

## Results

Uniform updated patient education handouts were created after comparing them with standard resources. A pre-test survey questionnaire was obtained to discuss with HCPs regarding the current knowledge and practices of the usage of patient education handouts and the understanding of EHR to utilize uniform and standardized patient education handouts. After educating the HCPs, their knowledge regarding the use of EHR to effectively use patient education handouts was tested in a post-test survey questionnaire. After completion of the pre and post-test survey questionnaire by HCPs, analysis of the data performed (Figures 1-20).

**Figure 1:**
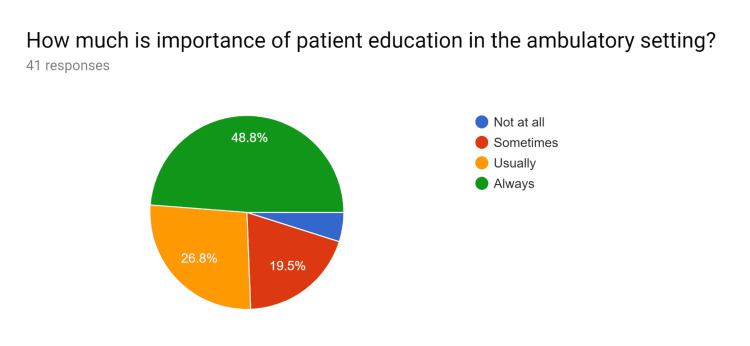
Pre-test survey responses (responses obtained prior to formal training of the HCPs) HCPs - healthcare providers

**Figure 2:**
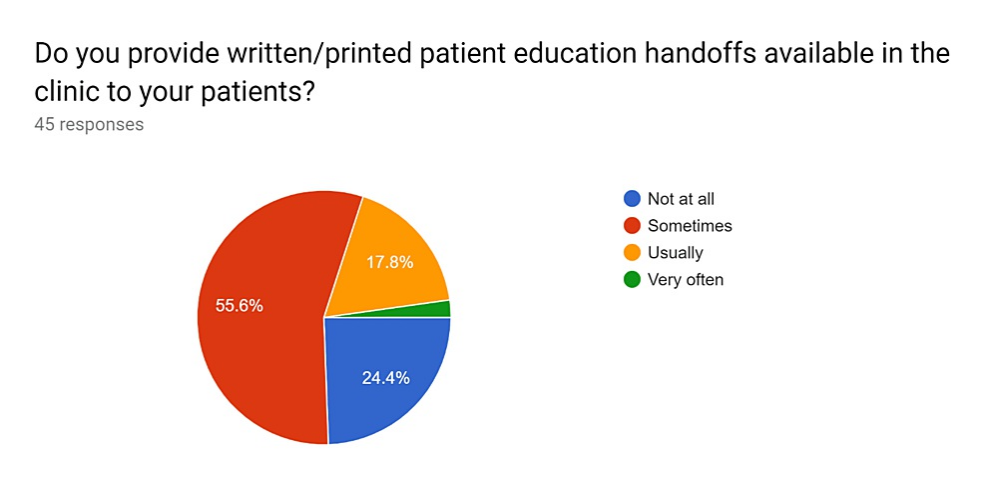
Pre-test survey responses (responses obtained prior to formal training of the HCPs) HCPs - healthcare providers

**Figure 3:**
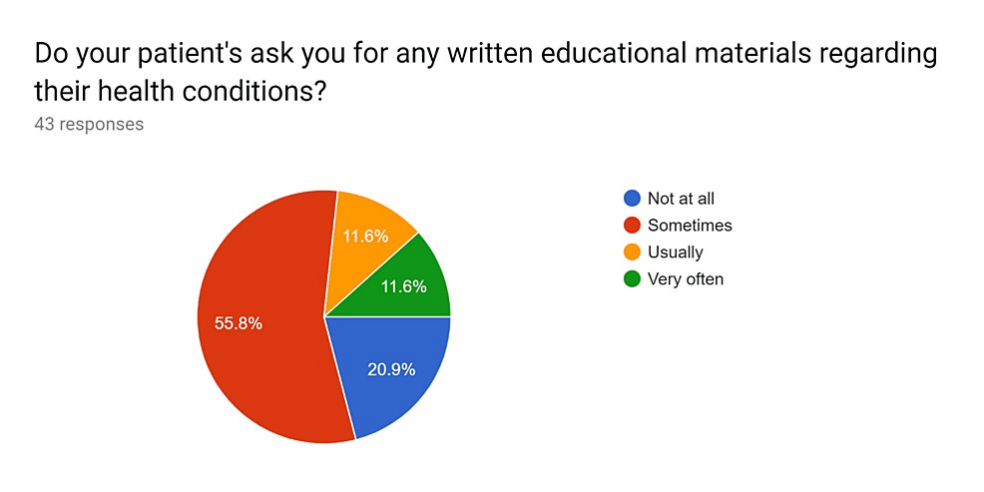
Pre-test survey responses (responses obtained prior to formal training of the HCPs) HCPs - healthcare providers

**Figure 4:**
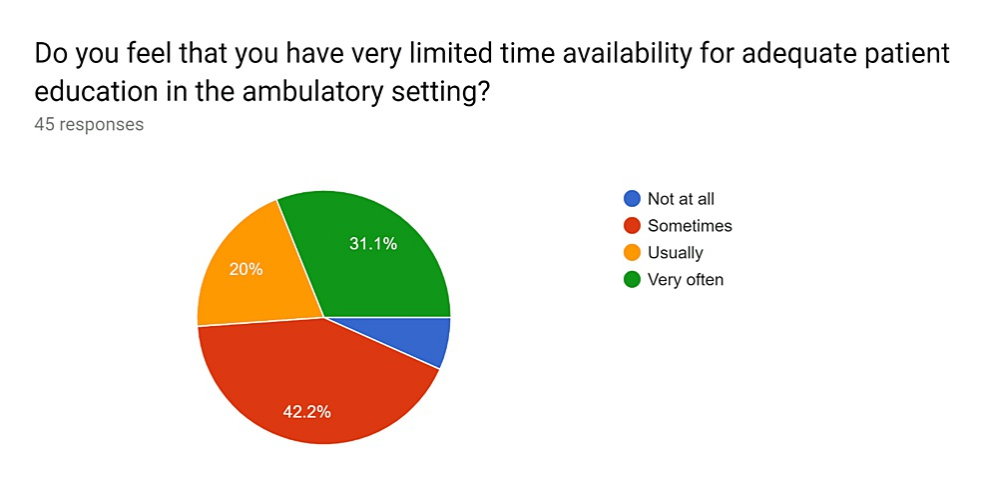
Pre-test survey responses (responses obtained prior to formal training of the HCPs) HCPs - healthcare providers

**Figure 5:**
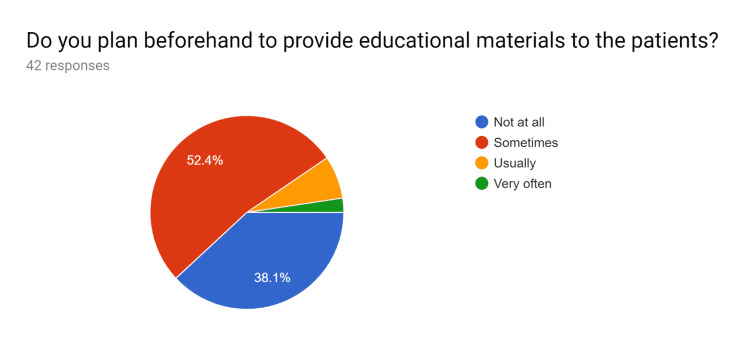
Pre-test survey responses (responses obtained prior to formal training of the HCPs) HCPs - healthcare providers

**Figure 6:**
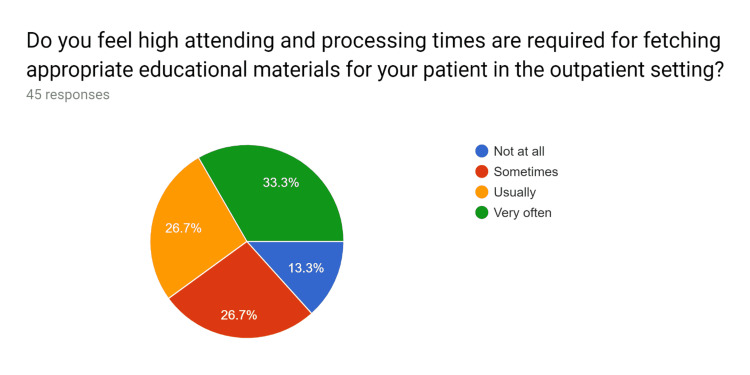
Pre-test survey responses (responses obtained prior to formal training of the HCPs) HCPs - healthcare providers

**Figure 7:**
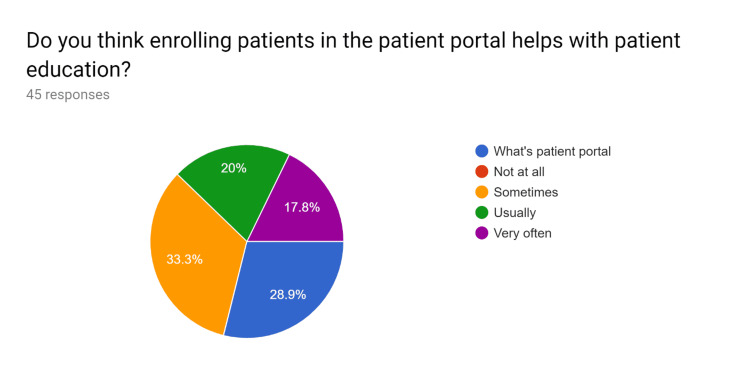
Pre-test survey responses (responses obtained prior to formal training of the HCPs) HCPs - healthcare providers

**Figure 8:**
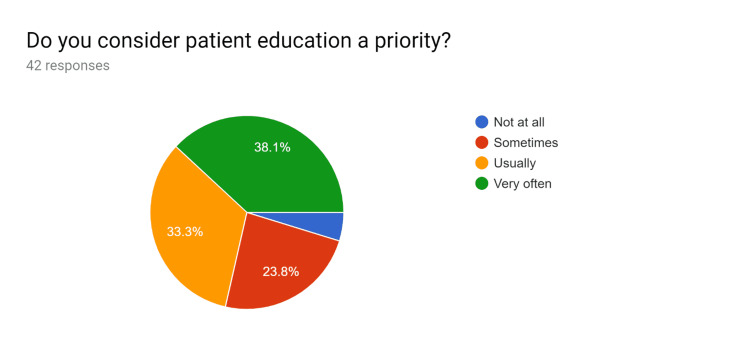
Pre-test survey responses (responses obtained prior to formal training of the HCPs) HCPs - healthcare providers

**Figure 9:**
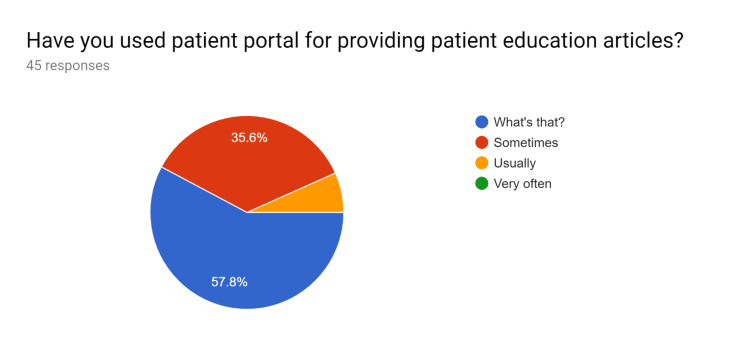
Pre-test survey responses (responses obtained prior to formal training of the HCPs) HCPs - healthcare providers

**Figure 10:**
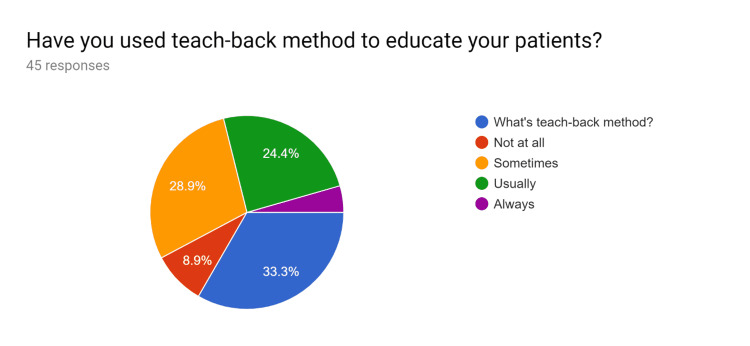
Pre-test survey responses (responses obtained prior to formal training of the HCPs) HCPs - healthcare providers

**Figure 11:**
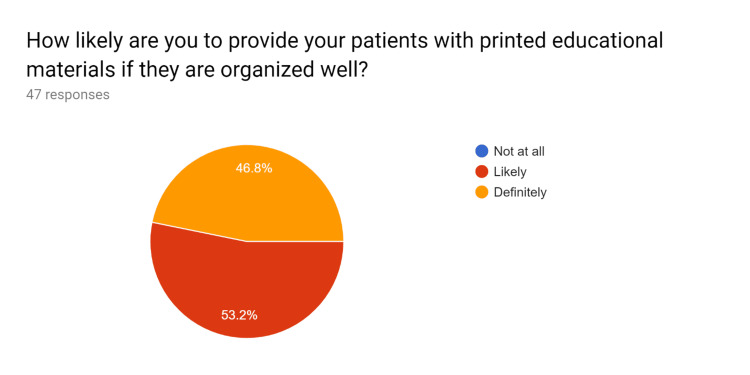
Post-test survey responses (responses obtained after formal training of the HCPs) HCPs - healthcare providers

**Figure 12:**
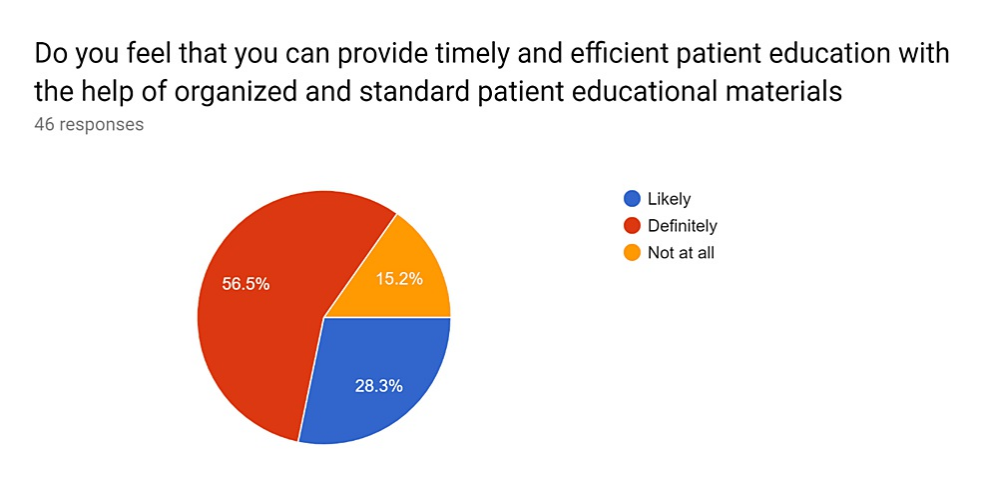
Post-test survey responses (responses obtained after formal training of the HCPs) HCPs - healthcare providers

**Figure 13:**
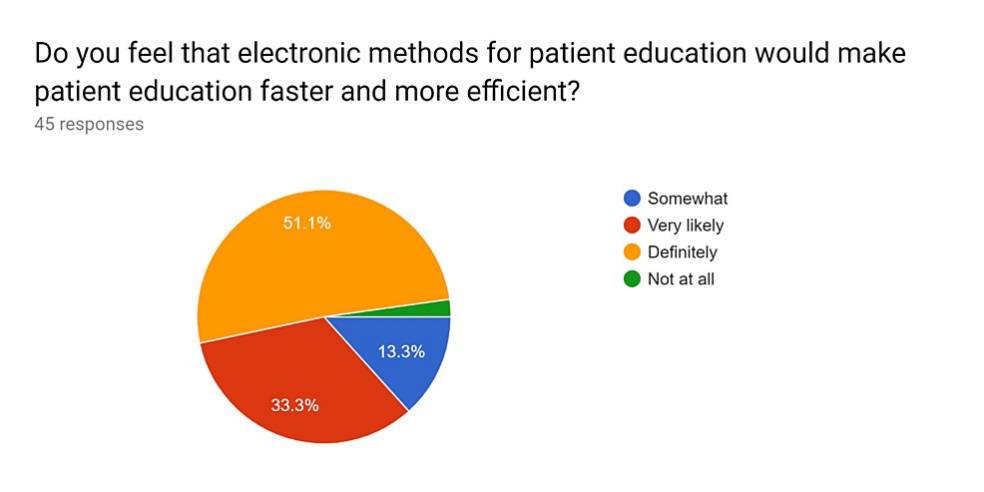
Post-test survey responses (responses obtained after formal training of the HCPs) HCPs - healthcare providers

**Figure 14:**
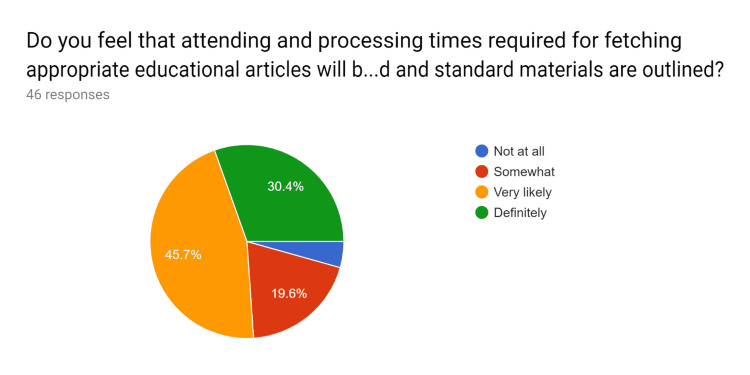
Post-test survey responses (responses obtained after formal training of the HCPs) "Do you feel that attending and processing times required for fetching appropriate educational articles will be reduced if standard materials are outlined?" HCPs - healthcare providers

**Figure 15:**
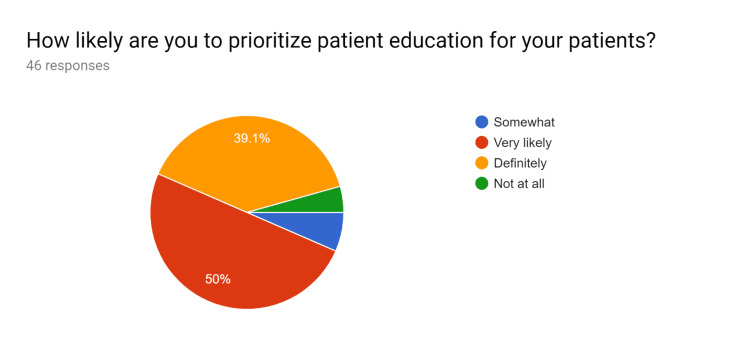
Post-test survey responses (responses obtained after formal training of the HCPs) HCPs - healthcare providers

**Figure 16:**
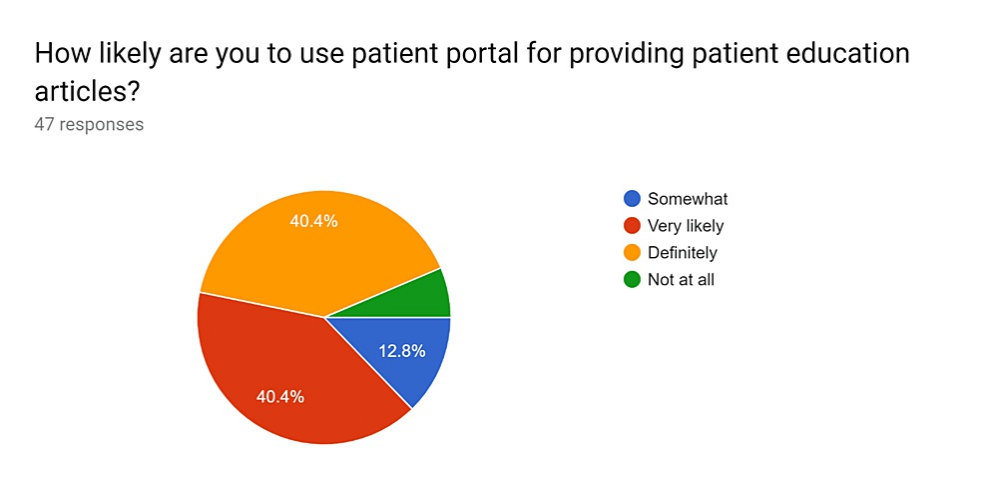
Post-test survey responses (responses obtained after formal training of the HCPs) HCPs - healthcare providers

**Figure 17:**
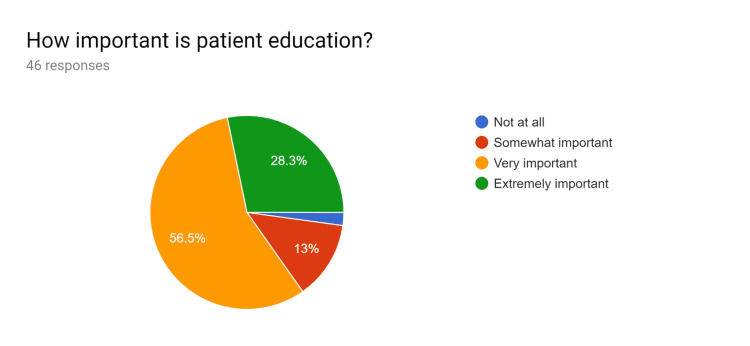
Post-test survey responses (responses obtained after formal training of the HCPs) HCPs - healthcare providers

**Figure 18:**
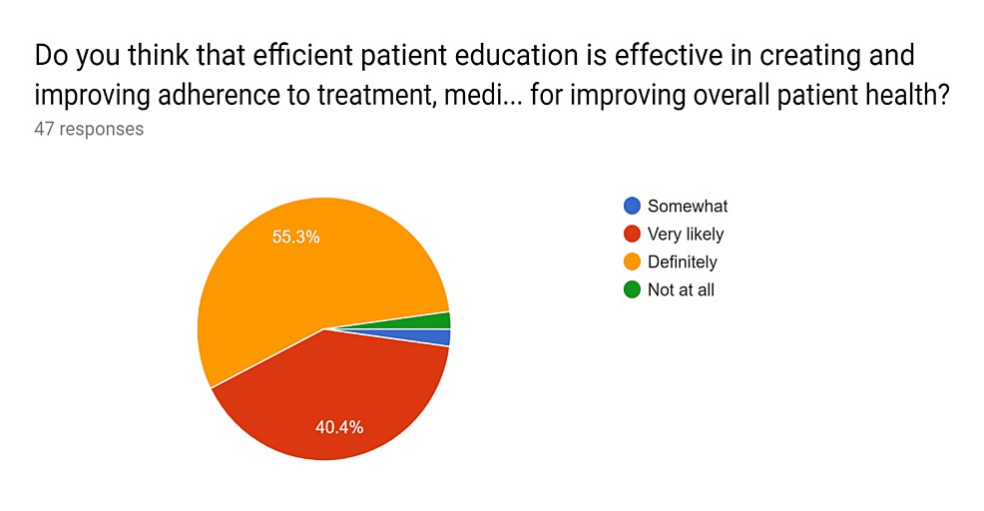
Post-test survey responses (responses obtained after formal training of the HCPs) “Do you think that efficient patient education is effective in creating and improving adherence to treatment, medication compliance, and for improving overall patient health?” HCPs - healthcare providers

**Figure 19:**
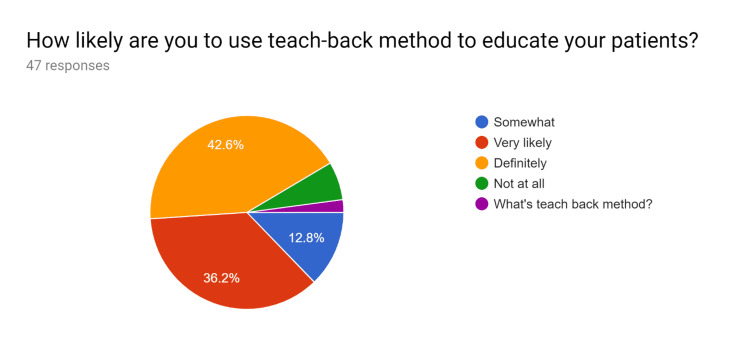
Post-test survey responses (responses obtained after formal training of the HCPs) HCPs - healthcare providers

**Figure 20:**
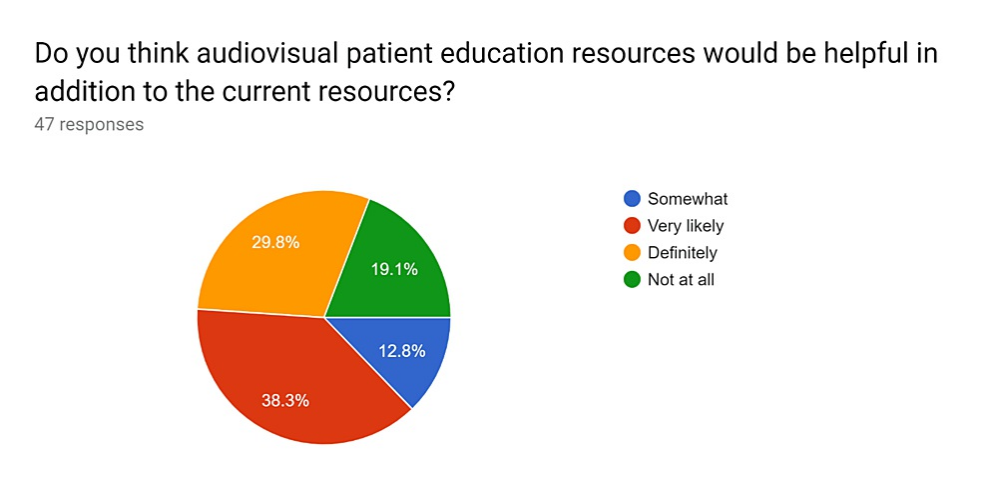
Post-test survey responses (responses obtained after formal training of the HCPs) HCPs - healthcare providers

Quality improvement (QI), problem-solving, and gap analysis

QI techniques, including PDSA cycles, to improve patient education implemented in various clinical settings [1].

Reasons for Action

There is a need for updated and uniform patient education materials in addition to verbal counseling of the patient to help them understand the disease condition, diagnostic studies, proper advice on medications, and when to call for help, thereby enhancing health literacy. There exists several patient education materials for various ailments, and the need to assess which one is better suited for a disease condition and contains concise information.

Initial State

We reviewed the available patient education material from the patient education database integrated in the EHR, and compared it with current standardized resources such as MedlinePlus, US National Library of Medicine of NIH, CDC, and the US Department of Health and Human Services. A thorough review of literature on patient education material was performed prior to starting the study.

Goal State

We compared more than one source regarding the appropriateness of patient education, most specifically, how to use the medications and when to call for help. The quality of educational materials regarding disease education, diagnostics education, education on medication use, and education on when to call for help was assessed. The resources described above were utilized for comparison.

Gap Analysis

**Figure 21:**
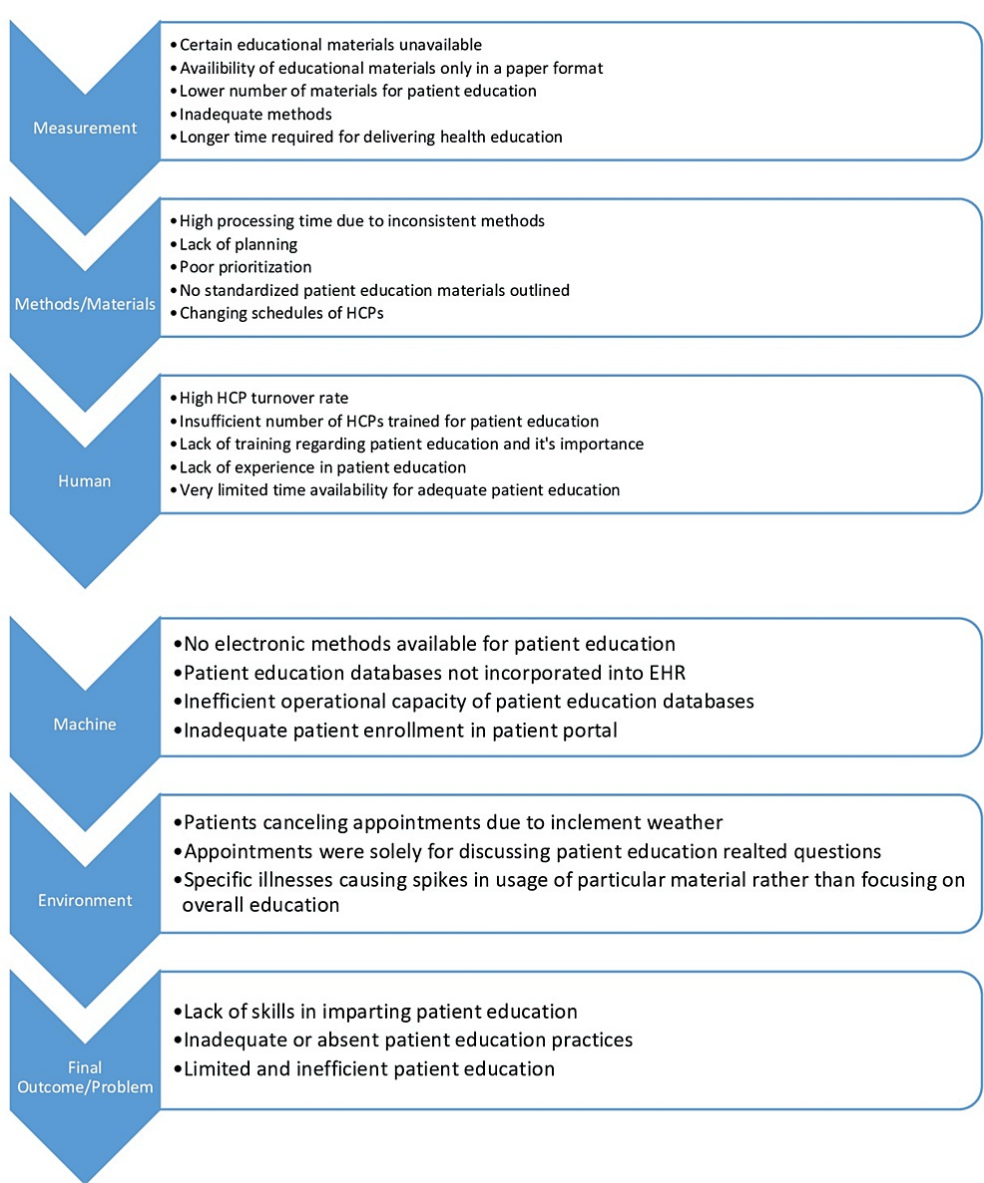
Gap analysis HCPs - healthcare providers

A graph of the gap analysis is displayed in Figure 21 below.

Solution Approach

It was noticed that the educational materials were available only in printed format. Enrolling patients on the electronic patient portal helps send educational materials to the patient as a soft copy in a faster and more efficient electronic format. 

Higher attending and processing time is required for fetching appropriate materials due to the unavailability of exact materials and using non-updated educational materials. Therefore, creating an index of educational articles on commonly encountered medical situations and ensuring that these articles are current and updated might make the process more efficient. 

There is a very limited time availability to impart specific educational elements with the limited appointment times. Appropriately detailed educational materials can be sent to the patient via a patient portal even after the patient encounter has ended. For patients with limited technology/computer use, educational materials can be mailed if they're missed during the encounter. 

Inadequate educational methods were utilized; thus, incorporating educational articles from resources other than the databases in the existing EHR, and using the index of educational articles on commonly encountered medical situations were applied.

Inefficient usage of the operational capacity of EHR for patient education, using database integrated in the EHR, and lack of training were identified. As a result, HCPs were trained on using educational materials for their patients in an efficient manner, and patient education was prioritized.

Rapid Experiment: Plan-Do-Study-Act Cycle

Plan: Plan to use appropriate patient education material from several sources made available in the index of the educational articles.

Do: Counsel and verbally educate the patients, along with providing educational materials. Obtain a verbal read-back from the patients about how to use medications and when to call for help.

Study: Use the teach-back method to make patients explain back the information provided in their own words to see if they understood the disease, diagnostics, medication use, and when to call for help to improve health literacy.

Act: If a patient has questions, address them appropriately and if need be, set up a follow-up appointment. 

Actions Taken

An index of educational materials relevant to the common medical ailments in various clinical settings was created. This index of educational materials was to guide HCPs in choosing appropriate and relevant articles in an efficient, quick, and timely manner for patients in various clinical settings. Effective use of patient educational materials in the database incorporated into the EHR, including electronic methods such as the use of the patient portal to help educate patients, was promoted. Alternate resources other than those from the database in the existing EHR were utilized. Educational materials in printed format were made available for patients with limited technology access. The amount of time required for fetching appropriate materials was reduced by creating and referencing to an index for commonly encountered medical situations.

Efficient and faster patient education was imparted with reduced processing and attending time required. Prioritized health education to improve health literacy. Efficient usage of operational capacity of database integrated in the EHR was undertaken to improve health literacy. HCPs were trained to use patient education materials efficiently. 

Insight

What Helped

Fast, efficient, and effective patient education helped patients and their families significantly in medical care and shared decision-making based on the most current and updated clinical evidence and patient preference. Creating an index of educational materials relevant to the medical conditions commonly encountered thereby reduced the amount of processing and attending time required for fetching appropriate materials. Effectively using patient educational materials in the database incorporated into the EHR, including electronic methods such as the use of a patient portal to help educate patients, using soft copy (electronic-copy) reduced requirement of printed materials. Correction of misconceptions that patients may have helped improve health literacy. 

What Went Well

Helping engage, encourage, and empower the patients in participating in their own health care and treatment decisions. Enhanced patient satisfaction and better outcomes (for instance, educating a patient on osteopenia encouraged them to continue/start the vitamin D supplementation, participate in regular exercise, healthy diet preferences, and health promotion). 

What Hindered

High HCP turnover rate with changing schedules hindered consistent use of patient education materials. Insufficient number of HCPs trained for patient education.

What Could Improve

Incorporating educational materials in the video format for patients who do not wish to read or talk about their health situations. Enhanced training of all the HCPs for effective and efficient use of patient education resources to allow consistency in effective patient education.

## Discussion

Personalized patient education engages, encourages, and empowers patients in participating in their own health care and treatment decisions and leading to better outcomes, decreased need for excess diagnostic testing, and enhanced patient satisfaction [3,4,5]. This needs motivation on the part of the resident doctors, nurse practitioners, physician assistants, physicians, and the allied staff. 

The Advisory Committee on Training in Primary Care Medicine (ACTPCMD) recommends that Health Resources & Services Administration’s (HRSA) Title VII, Part C, Section 747 and 748 education and training programs should prepare students, faculty, and practitioners to involve patients and caretakers in shared medical decision-making which can happen well with better patient education process [6].

We as HCPs should cultivate good habits amongst ourselves to ensure patients know about their condition and treatment well. This will help increase medication and treatment compliance amongst patients and enhance the physician-patient relationship to a higher level.

## Conclusions

To improve the physical and psychosocial well-being of a patient, personalized patient education materials, in addition to verbal education by the HCPs, augment the betterment of patient care via shared decision making and by improving patient satisfaction. There is a need to reiterate that HCPs understand patients' concerns and provide effective patient education and counseling for effective health care delivery.
